# Enhanced Resistance to *Sclerotinia sclerotiorum* in *Brassica rapa* by Activating Host Immunity through Exogenous *Verticillium dahliae* Aspf2-like Protein (VDAL) Treatment

**DOI:** 10.3390/ijms232213958

**Published:** 2022-11-12

**Authors:** Shufang Jiang, Weiwei Zheng, Zewei Li, Jingru Tan, Meifang Wu, Xinyuan Li, Seung-Beom Hong, Jianyu Deng, Zhujun Zhu, Yunxiang Zang

**Affiliations:** 1 Key Laboratory of Quality and Safety Control for Subtropical Fruit and Vegetable, Ministry of Agriculture and Rural Affairs, Collaborative Innovation Center for Efficient and Green Production of Agriculture in Mountainous Areas of Zhejiang Province, College of Horticulture Science, Zhejiang A&F University, Hangzhou 311300, China; 2 Department of Biotechnology, University of Houston Clear Lake, Houston, TX 77058-1098, USA; 3 College of Advanced Agricultural Sciences, Zhejiang A&F University, Hangzhou 311300, China

**Keywords:** *Brassica rapa*, *Sclerotinia sclerotiorum*, VDAL, immunity

## Abstract

Sclerotinia stem rot caused by *Sclerotinia sclerotiorum* is one of the most destructive diseases in *Brassica rapa*. *Verticillium dahliae* Aspf2-like protein (VDAL) is a secretory protein of *V*. *dahliae* which has been shown to enhance the resistance against fungal infections in several plants. Nonetheless, the molecular mechanisms of VDAL-primed disease resistance are still poorly understood. In this study, we performed physiological, biochemical, and transcriptomic analyses of *Brassica rapa* in order to understand how VDAL confers resistance to *S. sclerotiorumn* infections in plants. The results showed that foliar application of VDAL significantly reduced the plaque area on leaves inoculated with *S. sclerotiorum*. It also enhanced antioxidant capacity by increasing activities of superoxide dismutase (SOD), peroxidase (POD), peroxidase (APX), glutathione reductase (GR), protoporphyrinogen oxidase (PPO), and defense-related enzymes β-1,3-glucanase and chitinase during the infection periods. This occurred in parallel with significantly reduced relative conductivity at different periods and lower malondialdehyde (MDA) content as compared to sole *S. sclerotiorum* inoculation. Transcriptomic analysis showed a total of 146 (81 up-regulated and 65 down-regulated) differentially expressed genes (DEGs) in VDAL-treated leaves compared to the control. The most enriched three Kyoto Encyclopedia of Genes and Genomes (KEGG) pathways were the mitogen–activated protein kinase (MAPK) signaling pathway, plant hormone signal transduction, and plant-pathogen interaction, all of which were associated with plant immunity. DEGs associated with MAPK and hormone signal transduction pathways were ethylene response sensor ERS2, EIN3 (Ethylene Insensitive3)-binding F-box protein 2 (EBF2), ethylene-responsive transcription factor ERF94, MAPK 9 (MKK9), protein phosphatase 2C (PP2C37), auxin-responsive proteins (AUX/IAA1 and 19), serine/threonine-protein kinase CTR1, and abscisic acid receptors (PLY 4 and 5). Among the DEGs linked with the plant–pathogen interaction pathway were calmodulin-like proteins (CML5, 24, 27), PTI1-like tyrosine protein kinase 3 (Pti13) and transcription factor MYB30, all of which are known to play key roles in pathogen-associated molecular pattern (PAMP)-triggered immunity and effector-triggered immunity (ETI) for hypersensitive response (HR), cell wall reinforcement, and stomatal closure in plants. Overall, VDLA treatment triggered repression of the auxin and ABA signaling pathways and de-repression of the ethylene signaling pathways in young *B. rapa* seedlings to increase plant innate immunity. Our results showed that VDAL holds great potential to enhance fungal disease resistance in *B. rapa* crop.

## 1. Introduction

Chinese cabbage (*Brassica rapa* ssp. *Pekinensis*) is an economically important leafy vegetable crop that is cultivated worldwide, and it is rich in phytochemicals beneficial to human health [1]. Sclerotinia stem rot (SSR) disease caused by a broad-spectrum fungal phytopathogen *S. sclerotiorum* results in significant yield losses in *Brassica* species [2]. *S. sclerotiorum* is a typical necrotrophic fungus that manipulates the redox state of the host by secreting a non-specific phytotoxin oxalic acid (OA) and induces necrosis of plant tissues in order to obtain the nutrients [3]. Tissue necrosis results from an outburst of reactive oxygen species (ROS) at the site of infection, followed by apoptosis [4]. The regulation of OA-mediated suppression and induction of host ROS during the early and late stages of infection is an important mechanism of *S. sclerotiorum*-induced disease [5]. In addition to OA, the pathogenesis of *S. sclerotiorum* in *Brassica napus* was assumed to be associated with a peroxisome-related pathway, cell wall degradation, and detoxification of host metabolites [6].

To defend against microbial pathogens’ attack, plants have developed a complex array of innate immune systems that are activated when encountered with pathogen-derived molecules. They are typically classified into two categories. The first category is conserved molecular structures, referred to as pathogen-associated molecular patterns (PAMPs), which are perceived by receptor-like kinases (RLKs) and receptor-like proteins (RLPs) that function as specific pattern-recognition receptors (PRRs) localized at the plant cell surface [7,8]. Typical examples of PAMP and PRR pairs are flagellin peptide flg22 and its receptor FLS2, bacterial elongation factor EF-Tu peptide elf18 and its receptor EFR, and fungal cell wall component chitin and its receptor CERK1 [9]. PRRs, when interacting with the cognate PAMPs, activate a multitude of intracellular signaling pathways that ultimately lead to the acquisition of basal resistance [8,10]. This PRR-initiated immunity, also known as PAMP-triggered immunity (PTI), generally occurs upon the initial contact of non-adapted pathogens with plant cells, thus constituting the first layer of the immunity that stops the colonization of pathogens [11]. PTI occurs quickly upon the perception of PAMPs when pathogens infect plants and evokes a series of rapid physiological and biochemical responses to suppress pathogen invasion. [12,13,14,15,16]. PTI triggers early responses such as calcium flux and ROS burst, followed by intermediate responses including mitogen-activated protein kinase (MAPK)/calcium-dependent kinase (CDPK) activation, phytohormone signaling, and transcriptional reprogramming, and late responses such as stomata closure, callose, and lignin deposition [9,13,17,18,19,20,21]. The second category are race-specific effectors that are secreted by adapted microbial pathogens and delivered into plant cells through various secretion mechanisms. To counter the action of pathogen effectors, plants employ a second layer of immunity known as effector-triggered immunity (ETI) in which a host cytoplasmic receptor, called resistance (R) protein, recognizes an effector inside the plant cell and triggers disease resistance [22,23]. The R protein is typically a nucleotide binding site–leucine-rich repeat (NBS–LRR) protein that drives a series of biochemical reactions, culminating in a hypersensitive response (HR) in infected and surrounding cells or a systemic acquired resistance (SAR) in the whole plant system to limit further pathogen spread [23,24]. Although PTI and ETI use distinct receptors, they appear to use similar networks of calcium and ROS sensing, MAPK, and phytohormone signal transduction pathways and activate an overlapping set of genes for innate immunity [11,25,26].

The widespread use of pesticides to improve the yield and quality of agricultural products around the world has posed a considerable risk of environmental pollution, toxic poisonings to non-targeted organisms, and food safety [27,28]. In an attempt to protect the environment from the indiscrete use of agricultural chemicals, usage of environment-friendly natural products that can activate plant immunity, collectively known as elicitors or plant immunity inducers, has recently received much interest because of their ability to induce SAR in plants [29,30]. Plant immune inducers are also known as biostimulants; many researchers attach importance to them because of their environmental protection [31]. They represent a new field rapidly developing an approach to disease management, which includes plant immunity-inducing proteins, chitosan oligosaccharides, lipids, and microbes that can trigger defense responses and confer systemic resistance to subsequent pathogen attacks [32]. The first identified immunity-inducing protein was a so-called harpin produced by the fire blight bacterium [33], and it was permitted to be used commercially [34]. Since then, many other proteins have been discovered and studied: Pebc1 from *Botrytis cinerea* [35,36,37,38], PemG1 from *Magnaporthe grisea* [39], Hrip1 from *Alternaria tenuissima* [40,41,42,43], PeBA1 from *Bacillus amyloliquefaciens* [44], PeaT1 from *Alternaria tenuissima*, PeBC1 from *Brevibacillus laterosporus* [37,38], FocCP1 from *Fusarium oxysporum* [45], and AMEP412 from *Bacillus subtilis* [46,47]. These immunity-inducing proteins will eventually be used in agriculture and largely replace chemical pesticides in the future.

Similar to the above-mentioned pathogens, *V. dahliae* also secretes various proteins. *Verticillium dahliae* Aspf2-like protein (VDAL) is a class of effector proteins secreted by *V. dahliae*. Aspf2 is an allergen in *Aspergillus fumigatus* and homologous to Pra1, a zinc-binding protein secreted by *Candida albicans* [48]. Currently, many plant genes involved in *V. dahlia* resistance have been identified in a few plants [49,50]. VDAL was shown to enhance resistance to rice sheath blight as well as rice yield [48]. VDAL, when ectopically overexpressed in Arabidopsis or cotton, protected transcription factor MYB6 from its degradation by plant U-box 25 (PUB25) and PUB26 E3 ligases through interaction with them to enhance *Verticillium wilt* resistance without influencing growth and development [51]. Despite the VDAL-induced resistance against fungal infections in several plants, the effect of exogenous VDAL application on plant immunity against *S. sclerotiorum* in *B. rapa* is currently unknown. In this study, we performed a physiological, a biochemical, and a transcriptomic analysis to further investigate the molecular mechanism of VDAL-induced immune resistance to *S. sclerotiorum* in *B. rapa*. Our findings will help to understand the VDAL-induced immune resistance mechanism of *B. rapa* to provide a new strategy for plant disease control.

## 2. Results

### 2.1. Morphological Analysis of B. rapa Leaves after S. sclerotiorum Infection

**Figure 1:**
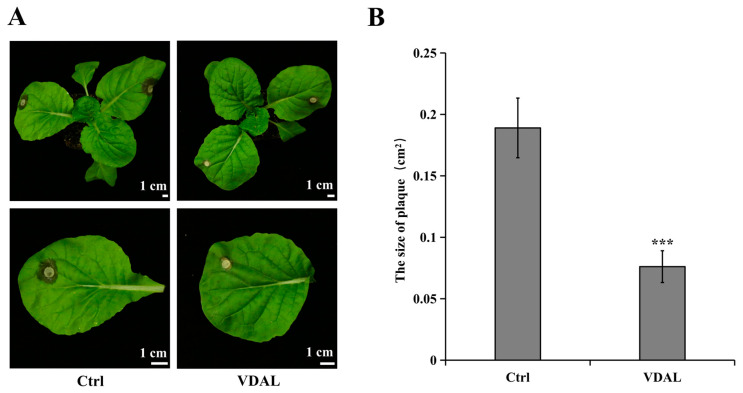
Effects of exogenous VDAL on *S. sclerotiorum* resistance in *B. rapa*. (**A**) Morphology and phenotype of representative *B. rapa* seedlings with water or VDAL treatment followed by *S. sclerotiorum* inoculation for one day. (**B**) Histogram of plaque area from leaves inoculated with *S. sclerotiorum* under water or VDAL treatment. Eighteen independent replicates were performed. Student’s *t*-test (*** *p* < 0.001) was used to estimate significant effects.

To study the effect of exogenous VDAL on *S. sclerotiorum* resistance, *B. rapa* leaves were inoculated with *S. sclerotiorum* after foliar application of water (Ctrl) or 1 g∙L^−1^ VDAL (VDAL). The plaque area of the leaves treated with 1 g∙L^−1^ VDAL was smaller than that of the control, suggesting that VDAL could improve the resistance of *B. rapa* against *S. sclerotiorum* (Figure 1).

### 2.2. Comparison of S. sclerotiorum Plaque Size on PDA Medium Supplemented with Water or VDAL

**Figure 2:**
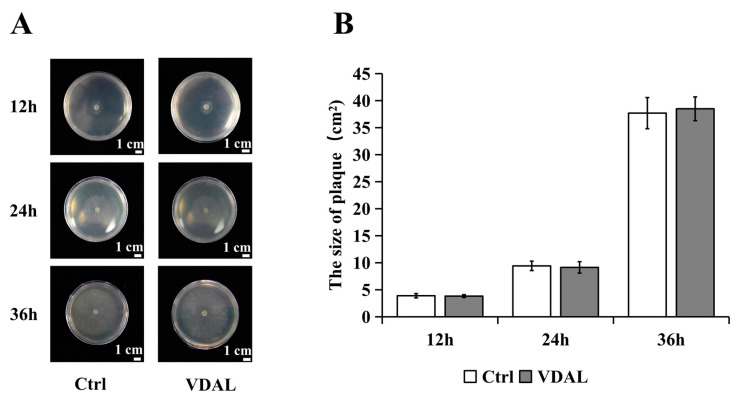
The effect of VDAL itself on *S. sclerotiorum*. (**A**) The plaque of *S. sclerotiorum* after being inoculated with water or VDAL on PDA medium after 12 h, 24 h, and 36 h, respectively. (**B**) Histogram of plaque area for *S. sclerotiorum* on PDA medium after water and VDAL treatment. Five independent replicates were performed. Student’s *t*-test was used to estimate significant effects.

To test whether VDAL can inhibit the growth of *S. sclerotiorum*, 5 mm mycelia were inoculated onto PDA medium in Petri dishes spread with water or 1 g∙L^−1^ VDAL. There were no significant differences between them (Figure 2), indicating that VDAL itself did not inhibit the growth of *S. sclerotiorum*.

### 2.3. Analysis of Antioxidant Enzyme Activities

**Figure 3:**
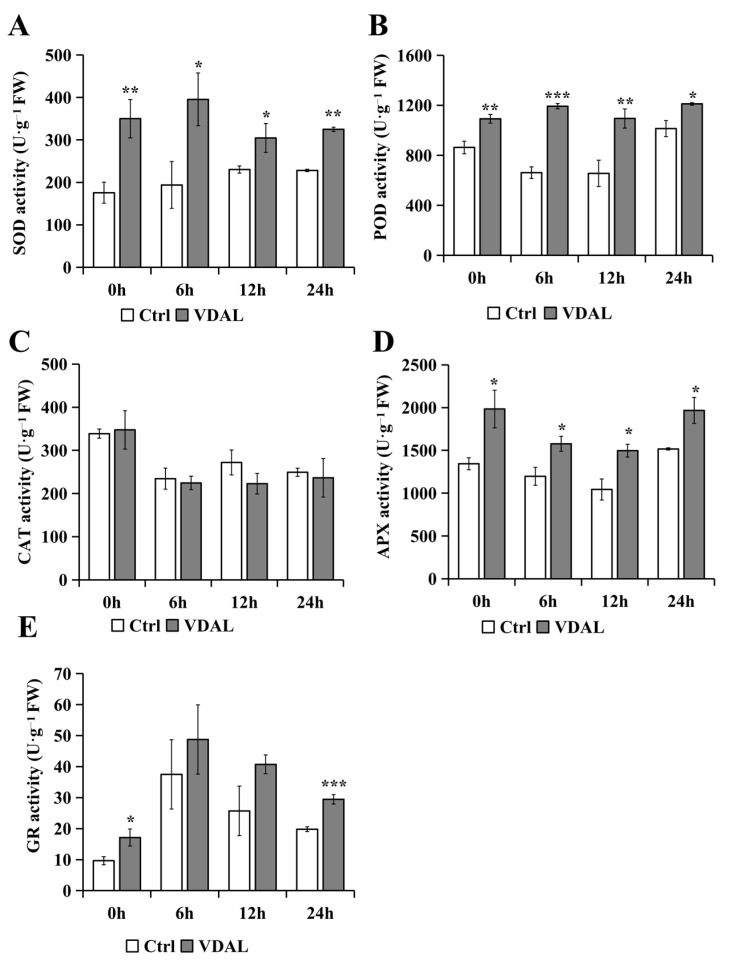
Antioxidant enzyme activities of *B. rapa* inoculated with *S. sclerotiorum* after spraying with water or 1 g∙L^−1^ VDAL. (**A**) SOD activity of *B. rapa* at 0, 6, 12 and 24 h after inoculation. (**B**) POD activity of *B. rapa* at 0, 6, 12, and 24 h after inoculation. (**C**) CAT activity of *B. rapa* at 0, 6, 12, and 24 h after inoculation. (**D**) APX activity of *B. rapa* at 0, 6, 12, and 24 h after inoculatioI (**E**) GR activity of *B. rapa* at 0, 6, 12, and 24 h after inoculation. Three independent replicates were performed. Student’s *t*-test (* *p* < 0.05; ** *p* < 0.01; *** *p* < 0.001) was used to estimate significant effects.

When plants are exposed to external stresses, antioxidant enzymes play a pivotal role in regulating ROS content [52]. Given this, we determined the activities of five antioxidant enzymes in *B. rapa* (Figure 3). The SOD activity of VDAL-treated *B. rapa* first significantly increased and then slightly decreased during the infection period, as shown by the 99.14%, 103.91%, 32.24%, and 42.53% increases at 0, 6, 12, and 24 h, respectively, compared to the control. (Figure 3A). On the other hand, the control showed statistically insignificant changes over time. Similar to SOD, the POD activity of *B. rapa* treated with VDAL was also significantly higher than that of the control at different stages of infection. During the infection period, the POD activity of VDAL-treated *B. rapa* remained relatively constant, while the control showed fluctuations (Figure 3B). However, VDAL treatment had no effect on the CAT activity of *B. rapa* throughout the infection time (Figure 3C). Notably, CAT activity significantly decreased at 6 h, after which its activity remained unchanged. Similar to the SOD and POD, the APX and GR increased significantly after treatment with VDAL (Figure 3D,E). However, APX activities in both treatments gradually decreased until 6 h and thereafter remained relatively unchanged (Figure 3D). The GR activity of VDAL-treated *B. rapa* was consistently higher than that of the control, and the GR activity of both treatments peaked at 24 h (Figure 3E). Collectively, VDAL increased the antioxidant capacity of *B. rapa*, thus contributing to resistance to *S. sclerotiorum.*

### 2.4. Analysis of General Defense-Related Enzyme Activities

**Figure 4:**
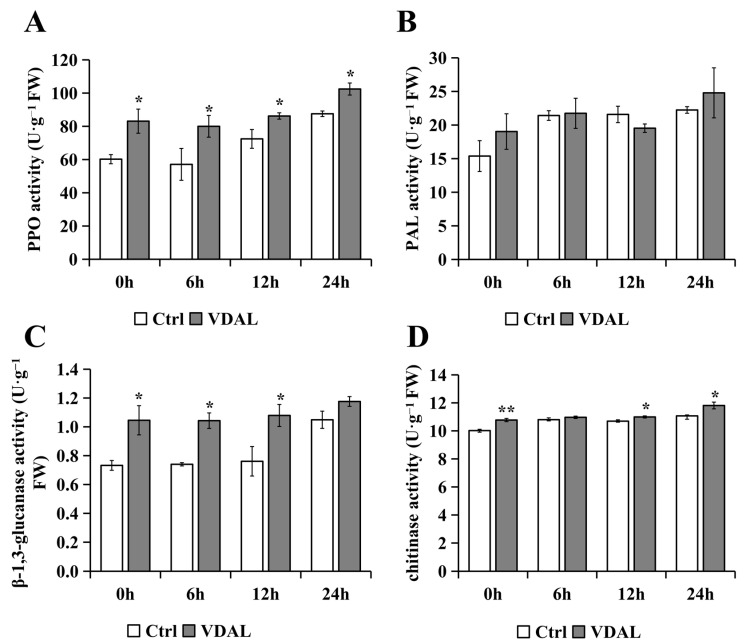
Defense-related enzyme activities of *B. rapa* inoculated with *S. sclerotiorum* after spraying with water or 1 g∙L^−1^ VDAL. (**A**) PPO activity of *B. rapa* at 0, 6, 12, and 24 h after inoculation. (**B**) PAL activity of *B. rapa* at 0, 6, 12, and 24 h after inoculation. (**C**) β-1,3-glucanase activity of *B. rapa* at 0, 6, 12, and 24 h after inoculation. (**D**) Chitinase activity of *B. rapa* at 0, 6, 12, and 24 h after inoculation. Three independent replicates were performed. Student’s *t*-test (* *p* < 0.05; ** *p* < 0.01) was used to estimate significant effects.

To find a correlation between VDAL priming and general defense-related enzyme activities in *B. rapa* seedlings, we measured the activities of four well-known enzymes: polyphenol oxidase (PPO), ammonia-lyase (PAL), β-1,3-glucanase activity, and chitinase. Compared to the control, the PPO activity of VDAL-treated *B. rapa* was significantly higher at different infection stages, peaking at 6 h (Figure 4A). VDAL had little effect on PAL activity in *B. rapa* (Figure 4B). β-1,3-Glucanase activity in VDAL-treated group increased by 42.70%, 40.88%, 41.77%, and 12.11% compared to the control at 0, 6, 12, and 24 h, respectively (Figure 4C). The activity level of β-1, 3-glucanase of VDAL-treated *B. rapa* remained unchanged throughout the infection stages, whereas the control exhibited a sharp increase at 24 h. Chitinase activity in VDAL-treated *B. rapa* was significantly higher than that of the control at all time points, except at 6 h (Figure 4D). Hence, the GR, PPO, β-1,3-glucanase, and chitinase, but not the PAL, were involved in the VDAL priming-mediated innate immunity of *B. rapa* seedlings.

### 2.5. Analysis of MDA, Relative Conductivity, and Soluble Sugar Content

**Figure 5:**
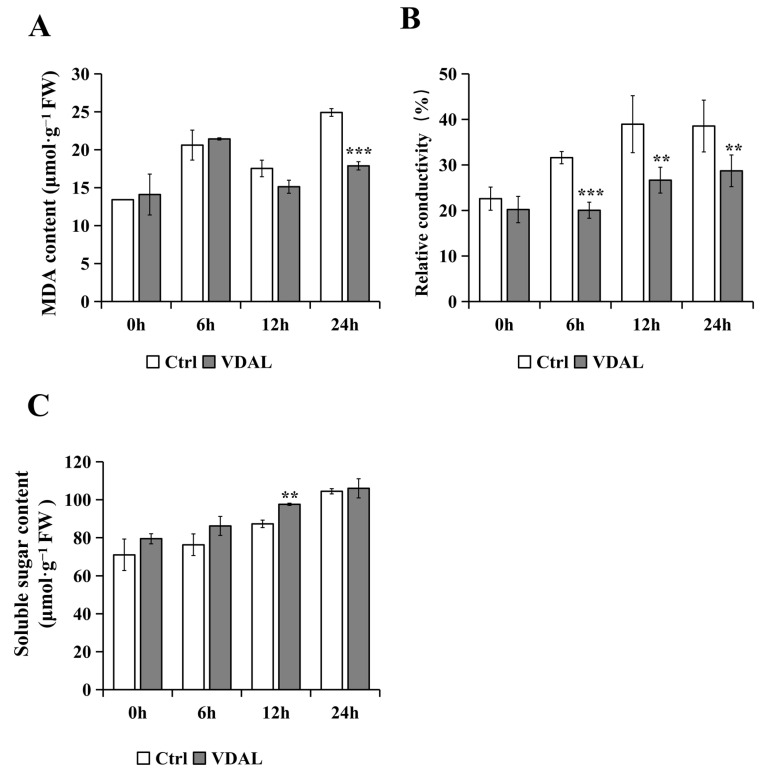
Injury related indicators of *B. rapa* inoculated with *S. sclerotiorum* after spraying with water or 1 g∙L^−1^ VDAL. (**A**) MDA content of *B. rapa* at 0, 6, 12, and 24 h after inoculation. (**B**) relative conductivity of *B. rapa* at 0, 6, 12, and 24 h after inoculation. (**C**) soluble sugar content of *B. rapa* at 0, 6, 12, and 24 h after inoculation. Three independent replicates were performed for MDA and soluble sugar. Six independent replicates were used for relative conductivity. Student’s *t*-test (** *p* < 0.01; *** *p* < 0.001) was used to estimate significant effects.

To further evaluate the antioxidant capacity of VDAL-primed *B. rapa*, MDA, and relative conductivity, which are widely used as a biomarker of oxidative stress and the antioxidant status, were examined. MDA contents in both treatments were not much different and showed a gradual increase from 0 to 6 h followed by a slow decrease from 6 to 12 h. After 12 h, the MDA content of VDAL-primed *B. rapa* rose only slightly, while that of the control rose to a noticeably higher level. Notably, the MDA content of VDAL-primed *B. rapa* was reduced at the highest level of 28.22% compared to the control at 24 h (Figure 5A). The relative conductivity of the two treatments gradually increased over a 12 h-infection time. The relative conductivity of the control was always significantly higher than that of the VDAL treatment after a 6 h infection (Figure 5B). Therefore, VDAL reduced the extent of membrane damage caused by *S. sclerotiorum*. Since a high level of sugar in plant tissues is known to promote the plant immune response against fungal pathogens [53], we measured soluble sugar levels in the two treatments. As shown in Figure 5C, soluble sugar contents of the two treatments successively increased during the infection, while there was no significant difference between the two treatments except for a 12 h infection time point. The reason for this transient increase in the 12 h VDAL treatment remains unclear. Overall, VDAL priming does not appear to increase sugars that may influence the expression of defense genes for *S. sclerotiorum* resistance in *B. rapa* seedlings.

### 2.6. Transcriptome Changes in Response to VDAL

To investigate the molecular responses of VDAL-induced *S. sclerotiorum* resistance, transcriptomic profiles were analyzed 24 h after *B. rapa* inoculation. RNA-Seq data from Ctrl and VDAL with three biological replicates were analyzed (Table S1). The base error rate was low at 0.02%, Q20%, and Q30% was over 98.10% and 94.52%, respectively, and the GC content was between 46.60% and 46.89%. A total of 291,011,900 raw reads were obtained from Ctrl and VDAL. After removing the low-quality reads and adapter sequences, 281,753,138 clean reads were obtained. The capacity of clean bases was 42.26 G. All clean data were then de novo assembled by StringTie (v1.3.3b), and assembly results were evaluated. A total of 281,753,138 Mb reads were obtained from six samples, with an average of 46,958,856 Mb reads in each sample (Table S2).

**Figure 6:**
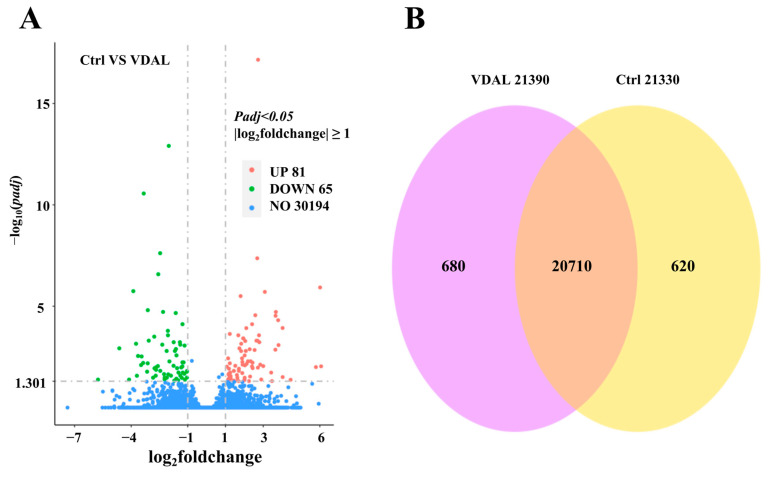
RNA-Seq data analyses. (**A**) Volcano plot of the overall DEGs distribution. The sum of read count values for each gene in the volcano map in different samples is greater than one. Genes with significant differences compared to the two groups were screened by threshold |log_2_ Fold Change| ≥ 1 and *Padj* ≤ 0.05. The X-axis depicts fold change in gene expression and the Y-axis depicts statistical significance. Red dots represent significantly up-regulated DEGs (UP), green dots represent significantly down-regulated DEGs (DOWN), and blue dots represent non-differentially expressed genes (NO). (**B**) The Venn diagram plots statistics for all expressed genes with FPKM > 1 in both VDAL and Ctrl. The pink area shows the genes expressed by VDAL, the yellow area shows the genes expressed by Ctrl, and the overlapping area shows the genes co-expressed by VDAL and Ctrl.

As compared to the control, the exogenous application of VDAL induced significant up-regulation of 81 genes and down-regulation of 65 genes (Figure 6A). Details of the DEGs are provided in Table S3. There were 22,010 genes with FPKM (fragments per kilobase of transcript per million mapped fragments) greater than one in both VDAL and Ctrl. Among them, 20,710 were co-expressed in VDAL and Ctrl, 680 were expressed only in VDAL, and 620 were expressed only in Ctrl (Figure 6B).

### 2.7. Gene Ontology (GO) and Kyoto Encyclopedia of Genes and Genomes (KEGG) Pathway Analysis

**Figure 7:**
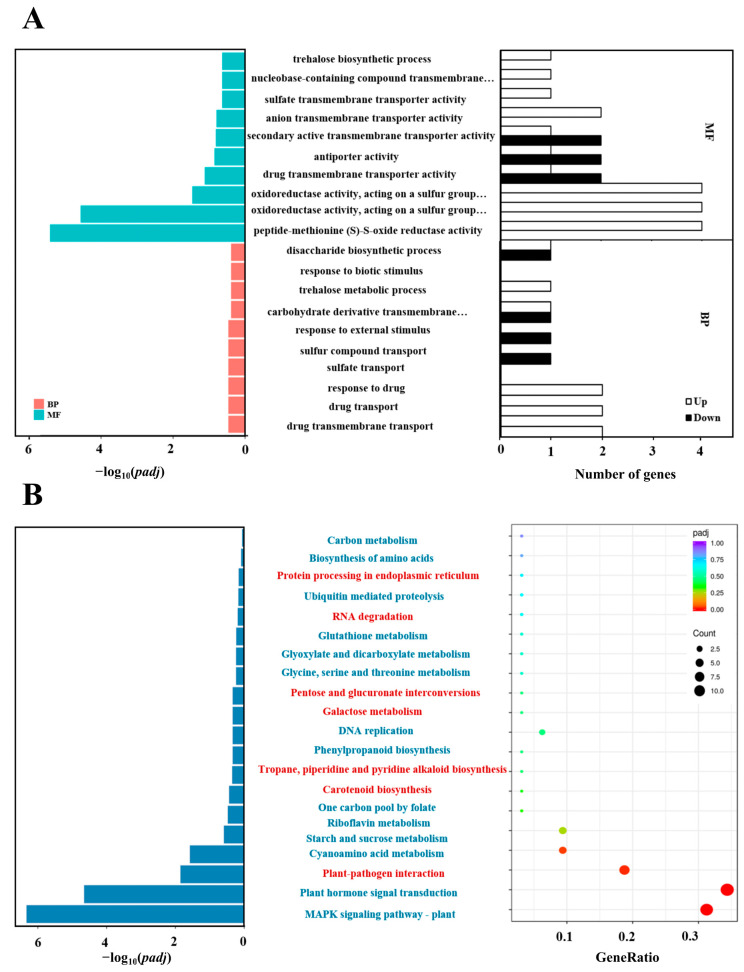
(**A**) GO enrichment analysis of DEGs. Bar graphs indicate the twenty most enriched GO terms (ranked by *p*-adjusted values) and the numbers of up- and down-regulated genes corresponding to the top ten GO terms in each of the two categories (BP: biology process, MF: molecular function). (**B**) Histogram presentation of the Kyoto Encyclopedia of Genes and Genomes (KEGG) classification of DEGs. Bar graphs indicate the 20 most significant enriched KEGG pathways (ranked by *p*-adjusted values). KEGG dot plot, the 21 biological processes with the largest gene ratios are plotted in order of gene ratio. The size of the dots indicated by ‘Count’ represents the number of genes in the significant DEGs list associated with the GO term, and the color of the dots represents the *p*-adjusted values. Gene ratio represents the ratio of the number of differentially expressed genes annotated to the KEGG pathway number to the total number of differentially expressed genes. The size of the dots represents the number of genes annotated to the KEGG pathway, and the color from red to purple represents the magnitude of enrichment.

To identify the function of sets of genes impacted by VDAL, we conducted a GO enrichment analysis of DEGs in VDAL relative to Ctrl. The GO function enrichment results were obtained based on the hypergeometric distribution principle and three biological domains of GO terms are biological processes (BP), molecular functions (MF), and cellular components (CC). Transcriptome analysis indicated that 30 BPs, 43 MFs, and 0 CC were involved in biological domains (Table S4). Twenty of the most enriched GO terms as well as their gene numbers were sorted out (Figure 7A). The top ten most enriched BPs were ‘drug transmembrane transport’ (GO:0006855), ‘drug transport’ (GO:0015893), ‘response to drug’ (GO:0042493), ‘sulfate transport’ (GO:0008272), ‘sulfur compound transport’ (GO:0072348), ‘response to external stimulus’ (GO:0009605), ‘trehalose biosynthetic process’ (GO:0005992), ‘trehalose metabolic process’ (GO:0005991), ‘response to biotic stimulus’ (GO:0009607), and ‘disaccharide biosynthetic process’ (GO:0046351). The top ten most enriched MFs were ‘peptide-methionine (S)-S-oxide reductase activity’ (GO:0008113), ‘oxidoreductase activity, acting on a sulfur group of donors, disulfide as acceptor’ (GO:0016671), ‘disulfide as acceptor, oxidoreductase activity acting on a sulfur group of donors’ (GO:0016667), ‘drug transmembrane transporter activity’ (GO:0015238), ‘antiporter activity’ (GO:0015297), ‘secondary active transmembrane transporter activity’ (GO:0015291), ‘anion transmembrane transporter activity’ (GO:0008509), ‘sulfate transmembrane transporter activity’ (GO:0015116), ‘nucleobase-containing compound transmembrane transporter activity’ (GO:0015932), and ‘carbohydrate derivative transmembrane transporter activity’ (GO:1901505). These data suggested that VDAL mainly regulated the activities of various transmembrane transporter and oxidoreductase of drug and sulfur-containing compounds.

KEGG is a resource and genome information base for systematic analysis of gene functions, providing information about genes and expression patterns from the perspective of the whole network [54]. Thus, KEGG analysis would provide information and further understanding of how VDAL activates the immune system of *B. rapa* protect against *S. sclerotiorum*. Based on the hypergeometric distribution principle, twenty-one of the most enriched KEGG pathways were selected, in which seven pathways contained relatively more up-regulated genes, and fourteen pathways comprised more down-regulated genes (Figure 7B). The three pathways with the most significant enrichment (*Padj* < 0.05) were the MAPK signaling pathway plant (brp04016), plant hormone signal transduction (brp04075), and plant–pathogen interaction (brp04626) (Table S5). There were one up-regulated and nine down-regulated genes involved in the MAPK signaling pathway, three up-regulated and eight down-regulated genes involved in plant hormone signal transduction, and six up-regulated genes in plant–pathogen interaction. The results suggested that VDAL primarily induced signaling pathways associated with plant disease resistance in *B. rapa*.

### 2.8. Gene Expression Analysis of Disease Resistance-Related Pathways

**Figure 8:**
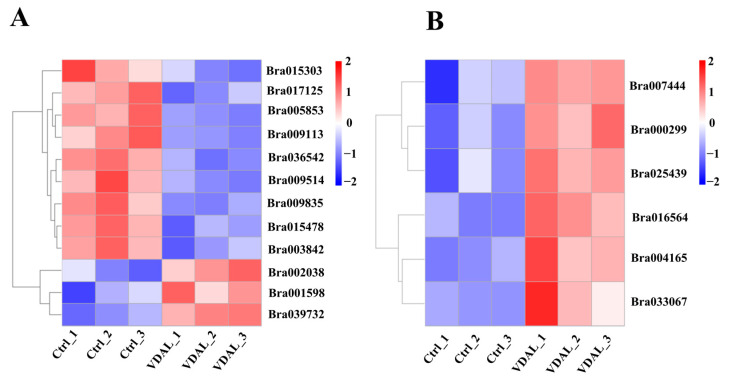
Heat map of hierarchical clustering of genes expressed in *B. rapa* inoculated with *S. sclerotiorum* after spraying with water or VDAL. (**A**) The heat map of genes associated with MAPK and plant hormone signal transduction. Bra015303, ethylene response sensor 2 (ERS2); Bra017125, abscisic acid (ABA) receptor PYL4; Bra005853, ABA receptor PYL5; Bra009113, ABA receptor PYL5; Bra036542, EIN3-binding F-box protein 2, Bra009514; serine/threonine-protein kinase CTR1; Bra009835, EIN3-binding F-box protein 2; Bra015478, ethylene-responsive transcription factor ERF094; Bra003842, mitogen-activated protein kinase kinase (MAPKK) 9; Bra002038, protein phosphatase 2C 37; Bra039732, auxin-responsive protein IAA1; and Bra001598, auxin-responsive protein IAA19. (**B**) The heat map of genes associated with plant–pathogen interaction. Bra007444, PTI1-like tyrosine-protein kinase 3 (Pti13); Bra000299, calmodulin-like protein 5 (CML5); Bra025439, calmodulin-like protein CML24; Bra016564, probable calmodulin-like protein CML27; Bra004165, calmodulin-like protein CML24; and Bra033067, transcription factor MYB30. The abscissa is the sample name, and the ordinate is the normalized value of the differential gene FPKM. The more intense the red color, the higher the expression level; the more intense the blue color, the lower the expression level.

All organisms rely on complex signaling networks to regulate their own metabolism and adaptation to the environment. To further explore the molecular responses triggered by VDAL action in more depth, we performed a heat map analysis of the DEGs in the three most enriched KEGG pathways of MAPK signaling, plant hormone signal transduction (Figure 8A), and plant–pathogen interaction (Figure 8B). All genes in Figure 8A, except genes of Bra003842, Bra039732, and Bra001598, co-regulated both the MAPK signaling pathway and plant hormone signal transduction pathway. Among twelve genes (Figure 8A), three genes were up-regulated by VDAL: Bra002038 encoding protein phosphatase 2C (PP2C), Bra039732, and Bra001598 encode auxin-responsive protein IAA1 and nineteen, respectively. The remaining down-regulated genes were ethylene response sensor, ethylene-responsive transcription factor, ABA receptors, EIN3-binding F-box proteins, serine/threonine-protein kinase, and MAPKK. On the other hand, all six genes linked with the plant–pathogen interaction pathway (Figure 8B) were up-regulated: Bra00744 encodes PTI1-like tyrosine-protein kinase 3; Bra000299 encodes calmodulin-like protein 5; Bra025439, Bra016564, and Bra004165 encoding calcium-binding proteins; and Bra033067 encodes transcription factor MYB30. The results indicated that VDAL activates the genes for calcium sensors and their downstream MAPK and phytohormone signaling pathways that trigger a plant immunity response.

### 2.9. Validation of Differential Expression by Quantitative RT-PCR

**Figure 9:**
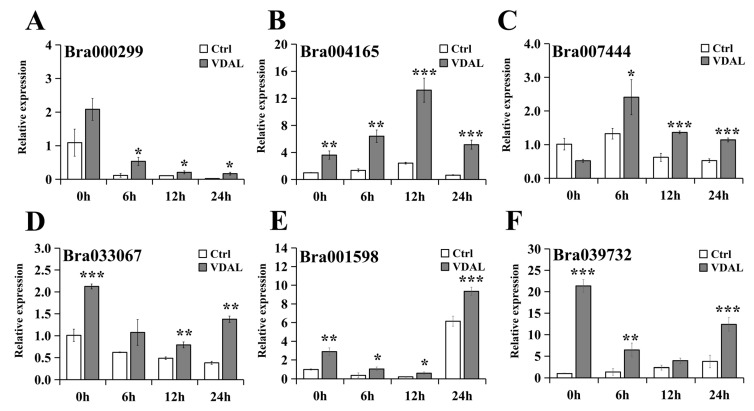
Expression levels of *B. rapa* defense-related genes relative to the control. *B. rapa* leaves were sprayed with water or 1 g∙L^−1^ VDAL. Shown are the relative expression levels of the Bra000299 (**A**), Bra004165 (**B**), Bra007444 (**C**), Bra033067 (**D**), Bra001598 (**E**), and Bra039732 (**F**) genes at 0, 6, 12, and 24 h after inoculation. Three independent replicates were performed. Student’s *t*-test (* *p* < 0.05; ** *p* < 0.01; *** *p* < 0.001) was used to estimate significant effects.

In order to validate our transcriptome data and investigate the mechanism underlying VDAL-triggered plant resistance, DEGs including Bra000299, Bra004165, Bra007444, Bra033067, Bra001598, and Bra039732 in the KEGG pathways related to disease resistance were selected for fluorescence quantitative verification (Figure 9). These DEGs were significantly up-regulated in our transcriptome analysis. Just as we expected, analysis of the fluorescence quantification results showed that the relative expression of DEGs was mostly higher in the VDAL treatment than in the control during the infestation. This independently confirmed that high coverage of transcriptome data is reliable.

## 3. Discussion

### 3.1. Exogenous VDAL Enhances Resistance to S. sclerotiorum by Stimulating the Antioxidant System

Plants have developed both enzymatic and non-enzymatic antioxidant defense systems to maintain the ROS equilibrium between the generation of ROS as redox signaling molecules, and the detoxification of ROS causing oxidative stress under harsh environmental conditions. The former include the SOD, POD, CAT, APX, and GR. Among them, the SOD removes O_2_^−^ and converts it to less reactive H_2_O_2_, while the CAT, POD, and APX reduce H_2_O_2_ to water [52]. The latter includes ascorbic acid (AsA), reduced glutathione (GSH), vitamins, and other secondary metabolites. GR is an NADPH-dependent oxidoreductase that plays a significant role in protecting cells against ROS by catalyzing the reduction of GSSG to GSH mediated by the AsA-GSH cycle with oxidation of NADPH, which results in efficient maintenance of the cellular reduced GSH pool [55]. In our study, *B. rapa* leaves sprayed with VDAL exhibited increased antioxidant capacity, as shown by higher activities of the SOD, POD, APX, and GR (Figure 3), and lower levels of MDA and relative conductivity than those of the control (Figure 5). Interestingly, no significant difference in CAT activity was observed between the control and VDAL treatment, and CAT activity decreased after 6 h of *S. sclerotiorum* infection (Figure 3C). This could be ascribed to steady-state intracellular levels of calmodulin (CaM)/Ca^2+^, salicylic acid (SA) and nitric oxide (NO), since the activity of plant CAT requires its binding to Ca^2+^/CaM [56], and SA and NO inhibit the activity of CAT [57,58]. Given the function of hydrogen peroxide as a key redox signaling molecule regulating gene expression and hypersensitive cell death [59,60], it would be important to sustain hydrogen peroxide homeostasis for plant survival under stress. The results, together with previous data, document that the activities of CAT, SOD, POD, APX, and GR antioxidant enzymes are coordinately regulated during development and differentially expressed depending on the type and intensity of stresses. Our KEGG analysis (Table S5) revealed that VDAL treatment led to the down-regulation of the gene (Bra036570) encoding gamma-glutamylcyclotransferase 2–1 that catalyzes GSH degradation to component amino acids L-glutamate, L-cysteine, and L-glycine [61]. This would further reinforce the GR-mediated GSH pool. Overall, exogenous VDAL stimulated the antioxidant system to enhance resistance to *S. sclerotiorum*, while maintaining intracellular ROS homeostasis.

### 3.2. Exogenous VDAL Enhances Resistance to S. sclerotiorum by Inducing General Defense-Related Enzyme Activities

It has been well documented that PPOs, PALs, chitinases, and β-1,3-glucanases are induced as part of a general defense mechanism in response to various plant pathogens [62,63,64]. PPOs are capable of oxidizing a broad spectrum of phenolic compounds into highly reactive quinones that could be toxic to invading pathogens [64]. The PAL catalyzes the first rate-limiting step in the phenylpropanoid pathway that produces a precursor for the lignin, flavonoid, anthocyanin, coumarin, SA, and a wide variety of other phenolic defense compounds possessing antioxidant and antimicrobial features [24]. The chitinases and pathogenesis-related (PR) group 2 β-1,3-glucanases are cell wall degrading enzymes that hydrolyze β-1,4-glycosidic and β-1,3-glycosidic bonds of β-glucans found in the cell walls of microbes, respectively, and plants can use these enzymes for a local antimicrobial defense barrier, as well as for generating signaling glucan molecules that triggering plant immune response [65,66]. Our study showed that the activities of PPOs, chitinases, and β-1,3-glucanases were substantially increased, whereas PAL activity was not affected by VDAL treatment (Figure 4). The lack of increased PAL activity could be related to a complex set of regulatory networks for developmental and environmental control of phenylpropanoid biosynthesis in which the PAL is not only transcriptionally regulated [67] but also prone to a rapid turnover by post-translational regulation via proteasome-mediated degradation by F-box proteins [68,69] and negative feedback regulation at metabolic level [70]. Hence, unlike cell wall degrading proteins, VDAL would not likely interfere with the multifaceted control mechanism of PAL that plays a key gateway role in mediating carbon flux from primary metabolism into secondary metabolism to increase plant fitness [63]. Instead of PAL gene activation by VDAL, PAL activity could be increased when plants were infected with *S. sclerotiorum*, making no significant difference between Ctrl and VDAL treatment. Along this line, it has been reported that the expression of many phenylpropanoid genes including PAL was induced upon pathogen attack [71,72]. The results, together with the previous data, suggest that VDAL may assist in fine-tuning the activities of defense-related enzymes to elicit a general defense mechanism.

### 3.3. Exogenous VDAL Enhances Resistance to S. sclerotiorum by Regulating Expression of the Genes Involved in MAPK and Hormone Signaling Pathway

**Figure 10:**
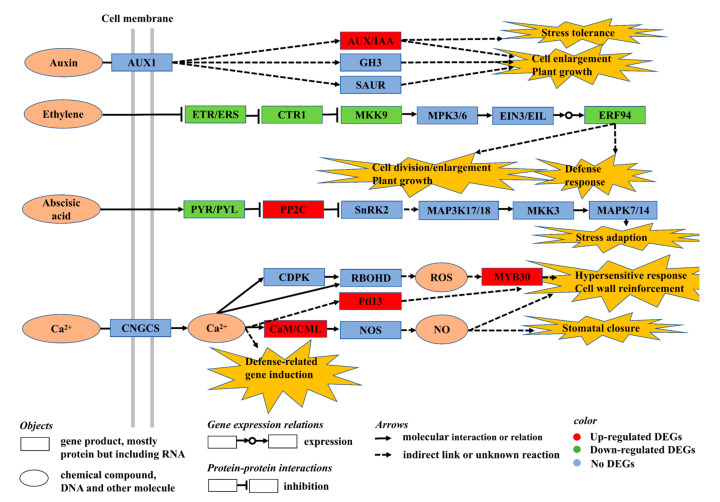
Metabolism and signaling pathway map. Abbreviations: *AUX1*, auxin transporter-like protein; *AUX/IAA*, auxin-responsive protein IAA; *GH3*, indole-3-acetic acid-amido synthetase GH3; *SAUR*, auxin-responsive protein *SAUR*; *ETR/ ERS*, ethylene receptor 1; *CTR1*, MAPKKK serine/threonine-protein kinase CTR1; *MKK9*, mitogen-activated protein kinase kinase 9; *MPK3/6*, mitogen-activated protein kinase 3/6; *EIN3/EIL*, ethylene-insensitive 3-like 3 protein; *ERF94*, ethylene-responsive transcription factor 94; *PYR/PYL*, abscisic acid receptor PYR/PYL; *PP2C*, protein phosphatase 2C; *SnRK2*, serine/threonine-protein kinase SRK2; *MAP3K17/18*, mitogen-activated protein kinase kinase kinase 17/18; *MKK3*, mitogen-activated protein kinase kinase 3; *MAPK7/14*, mitogen-activated protein kinase 7/14; *CDPK*, calcium-dependent protein kinase; *RBOHD*, respiratory burst oxidase homolog D; *CNGCS*, cyclic nucleotide-gated ion channel; *CaM/CML*, calmodulin and calmodulin-like protein; *NOS*, NO-associated protein 1; Pti13, PTI1-like tyrosine protein kinase 3; and MYB30, transcription factor MYB30. The signaling components indicated by the boxes filled with the red color were up-regulated by VDAL, whereas those denoted by the boxes filled with the green color were down-regulated by VDAL. The signaling components indicated by the boxes filled with the light blue color were not affected by VDAL.

Plant disease resistance is a complex cellular process involving a tradeoff balance between defense responses and plant productivity to achieve whole-plant adaptation and sustained growth. Phytohormones play critical roles in helping plants to adapt to diverse environmental conditions through the intricately interconnected signaling networks of growth-promoting and stress response hormones [73]. To assist in understanding the molecular responses evoked by VDAL action, we have presented a schematic signaling pathway map (Figure 10) based on the top three most enriched KEGG pathways of MAPK signaling, plant hormone signal transduction, and plant–pathogen interaction, as well as previous studies [74,75,76]. Auxin signaling commences with the auxin transporter-like protein (AUX1) that affects its three downstream three primary proteins of auxin-responsive protein IAA (AUX/IAA), indole-3-acetic acid-amido synthetase GH3 (GH3), and auxin-responsive protein SAUR (SAUR). Our KEGG analysis revealed that the genes encoding AUX/IAA1 and the 19 that are known to function as transcriptional repressors of early auxin response genes [77] were significantly up-regulated after VDAL treatment. This would result in the repression of the auxin signaling pathway that may lead to inhibition of cell division and elongation. Auxin signaling was shown to exert a negative effect on plant resistance to biotrophic pathogens, and thus repression of auxin signaling was assumed to be part of plant immune response [78,79]. It has been demonstrated that the auxin gene regulatory network interacts with the stress pathway through transcription of the *AUX/IAA* genes as hubs integrating signals from diverse pathways and that AUX/IAA19 is required for stress tolerance [80]. Thus, enhanced disease resistance would necessitate repression of auxin signaling that is integrated into the stress signaling pathway to strike a tradeoff balance between plant growth and stress responses.

In contrast to auxin, the genes involved in the ethylene signal transduction pathway, ethylene response sensor ERS2, serine/threonine-protein kinase CTR1 (CTR1), mitogen-activated protein kinase kinase 9 (MKK9), and ethylene-responsive transcription factor 94 (ERF94) were all significantly down-regulated. CTR1 is a Raf-like MAPKKK (mitogen-activated protein kinase kinase kinase) family protein that has been proposed to repress the autophosphorylation activity of MKK9 and MPK3/6 [81,82]. ERS2 is known to act as a redundant negative regulator of receptors-initiated ethylene signaling [83], and thus the down-regulated expression of *ERS2* would tend to derepress the ethylene signaling pathway. Once ethylene is sensed by receptors, the ethylene signaling and response pathway leading to the final downstream component ERFs is assumed to be transduced through the activity of antagonistic MAPK cascades, in which CTR1 initiates negative-acting MAPK cascades, whereas MKK9-MPK3/6 are positive-acting MAPK ones [82,84,85]. VDAL also down-regulated to *EBF2* that encodes ethylene insensitive 3 (EIN3)-binding F-box protein 2 as a component of SCF type E3 ubiquitin ligase complexes, which degrades EIN3/EIL1 transcription factors (TFs) in the nucleus to prevent the activation of ethylene-responsive genes [84,86,87]. Thus, reduced expression of EBF2 would enhance the accumulation of EIN3/EIL1, which culminates in the activation of stress response genes. Consequently, down-regulation of ERS2, CTR1, and EBF2 would facilitate a synergistic action for inducing ethylene signaling and response pathway. The role of ethylene under stress was shown to be dual by regulating a defense response, mostly in full-grown leaves, and a growth response in young leaves where ethylene impedes shoot growth by inhibiting cell division and cell expansion [88]. Hence, activation of the ethylene signaling pathway by VDAL would prioritize growth arrest in young *B. rapa* seedlings to increase plant fitness for a tradeoff between growth and defense responses. This interpretation is consistent with the aforementioned repression of the auxin signaling pathway by VDAL to redirect plant metabolism toward defense responses.

In addition, VDAL treatment down-regulated the genes encoding abscisic acid receptors PYL4 and 5 and up-regulated the gene encoding protein phosphatase 2C (PP2C), which is inhibited by PYR/PYL [89]. This type of co-regulation of ABA signaling components would lead to the suppression of ABA signaling and response pathway in which PP2C suppress serine/threonine-protein kinase SnK2 (SnRK2), followed by a series of cascade reactions involving mitogen-activated protein kinase kinase kinase 17/18 (MAP3K17/18), mitogen-activated protein kinase kinase 3 (MKK3) and mitogen-activated protein kinase 7/14 (MAPK7/14) [90,91]. The coordinated expression control of PLYs and PP2Cs by VDAL treatment, which would lead to a synergistic negative action for the ABA signaling pathway, likely enhances plant immunity response and resistance to the infection process due to a negative role of ABA in plant immunity of both PTI and ETI [92]. Collectively, the results indicated that VDLA treatment triggered repression of the auxin and ABA signaling pathways and de-repression of the ethylene signaling pathway in young *B. rapa* seedlings to increase plant fitness suitable for withstanding biotic stress caused by *S. sclerotiorum* infection.

### 3.4. Exogenous VDAL Enhances Resistance to S. sclerotiorum by Regulating Expression of the Genes Involved in the Plant–Pathogen Interaction Pathway

In plants, calcium signal transduction is involved in a wide range of cellular processes, from abiotic and biotic stress responses to development and growth. Both PTI and ETI are known to induce cytosolic Ca^2+^ elevations, in which PTI activation involves a transient Ca^2+^ peak that returns to basal levels within minutes, whereas ETI is associated with a prolonged cytosolic Ca^2+^ increase that can last for hours [76,93,94]. The increase in intracellular free Ca^2+^ levels may act as a specific signal that is translated into appropriate biological responses via downstream effectors capable of regulating different growth and developmental processes in response to various external stresses [95]. Thus, the specificity of molecular responses triggered by the VDAL effector could be partially dictated by calcium sensor proteins. In this study, four genes that encode calmodulin-like proteins, CML5 (Bra000299), CML24 (Bra025439, Bra004165), and CML27 (Bra016564) were significantly up-regulated by VDAL treatment. Although the subcellular locations and functional specializations of CML5 and 27 are currently unknown, CMLs are calcium-sensing proteins that regulate plant development and stress responses by relaying Ca^2+^ signals to the downstream target proteins for transcriptional responses, phosphorylation, or metabolic changes [96,97,98,99,100]. CMLs/Ca^2+^ were implicated to play an important role in the activation of plant immune responses [93,96,101]. CML24 is known to be expressed in all major organs in response to diverse stimuli and causes alterations in abscisic acid (ABA) levels and ionic stress [102]. In addition to calcium sensors, the genes for PTI1, such as tyrosine-protein kinase 3 (Pti13) and transcription factor MYB30, were also significantly up-regulated. Although specific target substrates of Pti13 have not been defined yet, Pti13 was identified as a putative *R* gene product that confers resistance to the soil-borne wheat mosaic virus [103] and leaf spot disease in peanuts [104]. MYB30 is a ROS-responsive R2R3-MYB transcription factor that regulates cytosolic Ca^2+^ concentration, as well as a positive regulator of the programmed cell death associated with HR through the activation of a very-long-chain-fatty-acid biosynthesis pathway [105,106,107,108]. MYB30 also was found to link RBOHD (respiratory burst oxidase homolog D)-driven ROS wave signaling to systemic acquired acclimation in Arabidopsis [109], indicating a close connection between the ROS and Ca^2+^ signaling pathways at the early stage in plant immunity. Accordingly, the concerted action of CML5/24/27, Pti13, and MYB30 would facilitate a rapid induction of plant innate immunity. According to the KEGG analysis (Table S5), the exogenous VDAL application down-regulated expression of the gene (Bra040301) encoding DNA replication licensing factor MCM2 as a component of the MCM2-7 complex that is essential for cell division [110], implicating the importance of cell cycle arrest in the process of VDAL-induced plant immunity. Taken together, the results strongly suggest that VDAL effector-induced cellular responses associated with the pathways of MAPK signaling, phytohormone signal transduction, and plant–pathogen interaction are coordinated and concerted for enhancement of plant innate immunity and cell cycle arrest by a complex, hierarchically organized set of regulatory networks that monitor and respond to the type of stresses.

## 4. Materials and Methods

### 4.1. Plant Materials and Experimental Treatments

*B. rapa* cv. ‘ZSN5’ was used for this experiment. Seeds were sown in soil and then cultivated for 26 days under well-regulated conditions with 65% relative humidity, 600 μmol∙m^−2^∙s^−1^ light intensity, 16 h/8 h, and 24 °C/20 °C, respectively, in day/night. VDAL (provided by China Agricultural University) was dissolved in water with a concentration of 1 g∙L^−1^. All solutions were prepared freshly before use. Seedlings with approximately five leaves were foliar sprayed with water or 1 g∙L^−1^ VDAL.

*S. sclerotiorum* was preserved at 4 °C and then reactivated in a Petri dish containing potato dextrose agar (PDA) medium (Becton Dickinson, Columbia, MD). The mycelium was inoculated into the center of the Petri dish using a 5 mm puncher. The Petri dishes were then incubated at 22 °C for 3 days to provide actively growing mycelium for leaf infection. New, marginal hyphae were excised by a puncher from the Petri dishes, which were then upended and closely arranged on the adaxial leaf surface 1 day after VDAL treatment. Those seedlings were then placed into incubation chambers with 90% humidity.

Leaf samples were harvested at 0 h, 6 h, 12 h, and 24 h post-infection with *S. sclerotiorum*. The plaque area was calculated by ImageJ software. Leaves inoculated with *S. sclerotiorum* were harvested, frozen in liquid nitrogen, and stored at −80 °C until later use. Three independent biological replicates were used for each experiment.

### 4.2. In Vitro Test of S. sclerotiorum on PDA Medium Sprayed with VDAL

Water or 1 g∙L^−1^ of VDAL was applied evenly on the PDA medium with a spreading stick. After even application, the mycelium with uniform growth was inoculated into the center of the Petri dish using a 5 mm hole punch. The Petri dishes were then placed in a constant temperature incubator at 22 °C. The growth of *S. sclerotiorum* in the Petri dishes was observed every 12 h.

### 4.3. Determination of Enzyme Activities

Activities of the superoxide dismutase (SOD), peroxidase (POD), and catalase (CAT) were determined using the kits R21883, R30312, and R22073 (Shanghai Yuanye Bio-Technology Co., Ltd., Shanghai, China), respectively. Activities of the glutathione reductase (GR), phenylalanine ammonia-lyase (PAL), polyphenol oxidase (PPO), and chitinase were determined using kits A062-1-1, A137-1-1, A136-1-1 and 139-1-1, respectively, (Nanjing Jiancheng Bioengineering Institute, Nanjing, China). Activities of the β-1,3-glucanase were measured using kits D799773 (Sangon Biotech Co., Ltd., Shanghai, China). The above enzyme activities were measured according to the instructions of the kits. The activity of the ascorbate peroxidase (APX) was determined as described previously [111]. Briefly, ground frozen leaf tissues (500 mg) were mixed with 5 mL of 50 mM potassium phosphate buffer containing 1 mM EDTA-Na_2_ (pH7.0), followed by centrifugation (15,000 r/min) for 15 min at 4 °C. The resultant supernatants were then employed as the enzyme extract for APX. The reaction mixture for APX activity was composed of 1.8 mL of 50 mM potassium phosphate buffer (pH7.0), 0.1 mL of 15 mM ascorbic acid, 100 µL of enzyme extraction solution, and 1 mL of 0.3 mM H_2_O_2_. Using a phosphate buffer as blank, the change of A_290_ was measured within 5 min. The decrease of A_290_ by 0.01 in 1 min was defined as 1 viability unit (1 U). The APX activity was calculated using the following formula: APX activity (U·g^–1^ FW) = ΔA_290_ × V_1_ / (0.01 × V_2_ × t × W), where ΔA_290_ is the amount of change in A_290_ during the reaction time; V_1_ is the total volume of extracted enzyme solution (mL); V_2_ is the volume of enzyme liquid used in the measurement. t is reaction time (min); and W is the fresh weight of the sample (g).

### 4.4. Determination of MDA, Soluble Sugar Content, and Relative Conductivity

MDA and soluble sugar content were determined by following the methods [112]. Briefly, 0.5 g of plant material and 5 mL of 10% trichloroacetic acid (TCA) were mixed, pulverized well, and the homogenate was centrifuged at 4000 rpm/min for 10 min at 4 °C. The resulting supernatant was used as a sample extract. To 2 mL of the extract, 2 mL of 0.6% thiobarbituric acid was added, mixed well, and reacted in a boiling water bath for 15 min. The reaction mixture was cooled rapidly and centrifuged (4000 rpm/min, 4 °C, 10 min). The absorbances of the supernatant were measured at 532 nm, 600 nm, and 450 nm. The MDA content was calculated using the equation: MDA (µmol·g^–1^ FW) = [6.45 × (A_532_ − A_600_) − 0.56 × A_450_] × V_r_ × V_t_/(V_s_ × W), where A_450,_ A_532_, and A_600_ are absorbance values at 450 nm, 532 nm, and 600 nm, respectively; V_r_: volume of reaction system; V_t_: total volume of extract; V_s_: volume of MDA extracted liquid; and W is the fresh weight of plant tissue. Soluble sugar content (µmol·g^–1^ FW) = 11.71 × A_450_× V_r_ × V_t_ / (V_s_ × W).

The relative conductivity was measured following the method [113]. The leaves were rinsed well with deionized water and were punched to make round leaves without the main leaf veins. An amount of 10 mL of deionized water was added to each tube containing 10 leaves, placed in a vacuum desiccator for 30 min, and shaken on an oscillator for 1 h. The tubes were shaken well, and the initial conductivity value S_1_ was measured with a conductivity meter. The tubes were placed in a boiling water bath for 10 min to extract all cell contents. The centrifuge tubes were removed, cooled to room temperature with tap water, and the final conductivity value S_2_ was measured. The relative conductivity is calculated according to the following formula: relative conductivity = S_1_/S_2_.

### 4.5. RNA Extraction, Library Construction, Sequencing and Bioinformatics Analysis

Three biological replicates were performed for both the control and VDAL treatment. Clean leaf tissues were wrapped in tin foil and immediately immersed in liquid nitrogen. The following experiments, including RNA extraction, library construction, sequencing, and bioinformatics analysis were commissioned by the Novogene Bioinformatics Technology Company (Beijing, China). The sequencing was conducted by the Illumina NovaSeq 6000. Clean data (clean reads) were obtained by removing reads containing adapter reads containing N base and low-quality reads from raw data. Meanwhile, all the downstream analyses including Q20, Q30, and GC content were calculated from the clean data. All the downstream analyses were based on clean data with high quality. A differential expression analysis of two conditions/groups (two biological replicates per condition) was performed using the DESeq2 R package (1.20.0). DESeq2 provides statistical routines for determining differential expression in digital gene expression data using a model based on the negative binomial distribution. The resulting *p*-values were adjusted using Benjamini and Hochberg’s approach for controlling the false discovery rate. *Padj* ≤ 0.05 and |log_2_ fold change| ≥ 1 were set as the threshold for significantly differential expression. Gene Ontology (GO) enrichment analysis of differentially expressed genes (DEGs) was implemented using the cluster Profiler R package (3.8.1), in which gene length bias was corrected. The GO terms with corrected *p*-value less than 0.05 were considered significantly enriched by DEGs. The Kyoto Encyclopedia of Genes and Genomes (KEGG) is a database resource for understanding high-level functions and utilities of the biological system (http://www.genome.jp/kegg/) (accessed on 20 November 2021). The Cluster Profiler R package was used to test the statistical enrichment of DEGs in KEGG pathways.

### 4.6. Quantitative Real-Time RT-PCR (qRT-PCR)

For qRT-PCR, total RNA was isolated using a Trizol kit R21086 (Shanghai Yuanye Bio-Technology Co., Ltd., Shanghai, China) and treated with RNase-free DNase to remove any genomic DNA contaminants. The purity of extracted RNA was assessed by the ratio of absorbance A_260_ to A_280_, using a NanoDrop ND-1000 spectrophotometer (Thermo Fisher Scientific Inc., Waltham, MA, USA), and mRNA was enriched by using the oligo(dT) magnetic beads from the total RNA. The high quality of the mRNA was reverse-transcribed into cDNA using a Prime Script TM RT reagent Kit R333-01 (Vazyme Biotech Co.,Ltd., Nanjing, China).

Quantitative triplicate RT-PCR was carried out using Taq Pro Universal SYBR qPCR Master Mix Q712-02 (Vazyme Biotech Co.,Ltd., Nanjing, China) on the CFX connect TM real-time PCR detection system (Analytik, Jena, German). The housekeeping gene *BrActin* was used as a reference gene for quantitative validation of the expression data. The gene-specific primers for the arbitrarily selected six DEGs from KEGG analysis are listed in supplementary material Table S6. The PCR cycling conditions comprised an initial polymerase activation step at 95 °C for 30 s, followed by 40 cycles of 95 °C for 10 s, and 60 °C for 30 s, and concluded by one cycle of 95 °C for 15 s, 60 °C for 60 s, and 95 °C for 15 s. After each PCR run, a dissociation curve was designed to confirm the specificity of the product and to avoid the production of primer dimers. The 2^−ΔΔCT^ method [114] was used to calculate the fold change of transcript level. Data were analyzed from three independent sets of biological replicates.

### 4.7. Statistical Analysis

Graphs were drawn using Excel 2019 (Microsoft, Redmond, WA, USA). Data were expressed as mean ± standard error, calculated by SPSS 20.0 analysis software (IBM, Chicago, IL, USA), and assessed using analysis of variance to determine statistical significance. Student’s *t*-test (* *p* < 0.05; ** *p* < 0.01; *** *p* < 0.001) was used to estimate significant effects. Photographs were edited in Microsoft PowerPoint 2019.

## 5. Conclusions

In the present study, we demonstrated that exogenous VDAL triggered significant physiological, biochemical, and transcriptional changes in the immunity response to *S. sclerotiorum in B. rapa* seedlings. In the process of activating plant immunity, various antioxidant and defense-related enzyme activities were significantly increased by VDAL application. VDAL treatment enhanced the expression of CML5/24/27, Pti13, and MYB30, all of which play key roles in the early stage of plant innate immunity. This was accompanied by the de-repression of the ethylene signaling pathway and repression of the auxin and ABA signaling pathways while inhibiting cell division. The results clearly indicated that VDAL triggered the orchestrated changes in the upstream calcium sensing and hormonal signaling cascade network outputs leading to the transcriptional reprogramming of downstream gene expression for the physiological and biochemical responses against pathogen infection. VDAL-induced transcriptomic, proteomic, and metabolic profiles may greatly differ depending on the stage of plant growth and development. More detailed investigations into the impacts of exogenous VDAL on *B. rapa* plants at different stages of vegetative growth are needed to gain a holistic view of flexible and dynamic defense processes at the molecular, physiological, and metabolic levels, thus helping to provide a comprehensive strategy for improving resistance to *S. sclerotiorum* infection.

## Data Availability

The data presented in this study are available in this manuscript.

## Supplementary Materials

The following supporting information can be downloaded at: https://www.mdpi.com/article/10.3390/ijms232213958/s1.
